# Food-Accessories; Their Influence on Digestion

**Published:** 1886-05

**Authors:** 


ARTICLE VI.

### FOOD-ACCESSORIES; THEIR INFLUENCE ON DIGESTION.

The results of experimental inquiries on the subject of foods and food-digestion, when scientifically conducted, cannot help being of great practical importance to man, so intimately is his perfection and intellectual activity dependent upon his ailmentation. Among the results of certain experiments on this subject by Sir W. Roberts, as given in the *Nineteenth Century*, the following will be found of interest.

Man, as the author says, is a very complex feeder; he has departed, in the course of his civilization, very widely from the monotonous uniformity of diet observed in animals in the wild state. Not only does he differ from other animals in cooking his food, but he adds to his food a greater or less number of condiments for the purpose of increasing its flavor and attractiveness; but, above and beyond this, the complexity of his food-habits is greatly increased by the custom of partaking, in considerable quantity, of certain stimulants and restoratives, such as tea, coffee, cocoa, and the various alcoholic beverages, which have become essential to his social comfort, if not his physicial well-being.

But the generalized food-customs of mankind are not to be viewed as random practices adopted to please palate or gratify our idle or vicious appetite. These customs must be regarded as the outcome of profound instincts, which correspond to important wants of the human economy. They are the fruit of colossal experience, accumulated through successive generations. They have the same weight and significance as other kindred facts of natural history, and are fitted to yield to observation and study lessons of the highest scientific and practical value.

First, with respect to the action of ardent spirits on digestion, experiments were made with "proof spirit," and with brandy, Scotch whiskey and gin; and the conclusion is, that, so far as salivary digestion is concerned, these spirits, when used in moderation and well diluted, as they usually are when employed dietetically, ra ther promote than retard this part of the digestive process; and this they do by causing an increased flow of saliva. The proportion must not, however, much exceed five percent; and gin seems to be less injurious than brandy or whiskey. It was noticed in these experiments that both of these interfered with the digestive process, precipitating the starch more readily, altogether out of proportion to the amount of alcohol they contained, and brandy was worse than whiskey; and this circumstance appears to be due to certain ethers and volatile oils in them; and brandy contains a trace of tannin, which has an intensely retarding influence on salivary digestion. Even very small quantities of the stronger or lighter wines—sherry, hock, claret and port—exercise a powerful retarding influence on salivary digestion. This is due to the acid—not the alcohol—they contain, and if this acid be neutralized, as it often is in practice, by mixing with the wine some effervescent alkaline water, the disturbing effect on salivary digestion is removed.

In the case of vinegar. it was found that 1 part in 5,000 sensibly retarded this process, proportion of 1 in 1,000 rendered it very slow, and of 1 in 500 arrested it completely; so that, when acid salads are taken with bread, the effect of the acid is to prevent any salivary digestion of the latter—a matter of little moment to a person with vigorous digestion but to a feeble dyspeptic one of some importance. There is a very wide-spread belief that drinking vinegar is an efficacious means of avoiding getting fat; and this belief would appear, from these experimental observations, to be well founded. If the vinegar be taken at the same time as farinaceous food, it will greatly interfere with its digestion and assimilation.

Effervescent table-waters, if they consist simply of pure water charged with carbonic acid, exercise a considerable retarding influence on salivary digestion; but if they also contain alkaline carbonates, as most of the table-waters of commerce do, the presence of the alkali quite removes this retarding effect.

With regard to "peptic" digestion. the results are still more surprising. It was found that with ten per cent. and under, of proof-spirit, there was no appreciable retardation, and only a slight retardation with twenty per cent; but with large percentages it was very different, and with fifty per cent. the digestive ferment was almost paralyzed. It was also observed that the weaker forms of alcoholic drinks (wine and beer) differed greatly in the influence on peptic digestion from that of the distilled spirits. They retarded it altogether out of proportion to the quantity of alcohol they contained. Port and sherry exercised a great retarding effect. Even in the proportion of twenty per cent, sherry trebled the time in which digestion was completed. It should further be borne in mind that this wine also retards greatly salivary digestion. Sherry, then, is injurious for persons of feeble digestive powers. With hock, claret and champagne, it was also ascertained that their retarding effect on digestion was out of proportion to the alcohol contained in them; but champagne was found to have amarkedly less retarding effect than hock and claret, due apparently to the mechanical effects of its effervescent qualities. The quantity of claret and hock often consumed by many persons at meals must exercise a considerable retarding effect on peptic digestion; but small quantities of these wines (and even sherry) may not produce any appreciable retarding effect, but act as pure stinulants.

With regard to malt liquors it was observed, as with wines, that they retarded peptic digestion in a degree altogether out of proportion to the amount of alcohol contained in them: and, when taken in large quantities, they must greatly retard the digestion, especially of farinaceous food.

Tea, coffee and cocoa were found to exert varying degrees of influence on the salivary digestion. The medium strength of the tea usually drank is estimated at four to five per cent; strong tea may contain as much as seven per cent; weak tea, as little as two per cent. Medium coffee has a strength of about seven per cent, and strong coffee twelve to fifteen per cent; cocoa, on the other hand, is generally weaker, not more than two per cent, and this may be one reason why it is more suitable to persons with feeble digestions than tea or coffee. Tea exercises a powerful inhibitory effect on salivary digestion, and this appears to be entirely due to the large quantity of tannin it contains; and, in order to diminish as far as possible its retarding influence on salivary digestion, it should be made weak and used sparingly, and it should not be taken with, but after the meal. Coffee, unless taken in very large quantity, has very little retarding effect on salivary digestion; this is explained by the fact that the tannin of tea is replaced in coffee by a substance called caffeo-tannic acid. Cocoa resembles coffee, and has little or no effect on salivary digestion; the use of coffee or cocoa is therefore preferable to that of tea for persons of feeble digestion.

With respect to the influence of tea and coffee on stomach digestion, it was found that they both exercised a remarkable retarding effect. There was no appreciable difference in the two beverages if they were of equal strength; but, as coffee is usually made of greater percentage strength than tea, its effect must ordinarily be greater. Cocoa also had much the same effect if used of the same strength as tea or coffee; but when of the strength ordinarily employed, its effect was inconsiderable. Strong coffee–*cafe noir*–had a very powerful retarding effect, and persons of weak digestion, should avoid the customary cup of "black coffee" after dinner.

Perhaps one of the most unexpected results of these experiments was the discovery that beef-tea had a powerful retarding effect on peptic digestion, as much so as that of a five per cent. infusion of tea. Further researches appear to show that this retarding effect of beef-tea was due to the salts of the organic acids contained in it. Beef-tea contains but very little nutritive properties, and must therefore be looked upon rather as a stimulant and restorative than as a nutrient beverage, but it is nevertheless very valuable on account of those properties.

The author holds the view that, in healthy and strong persons, the retarding effect on digestion, observed to be produce by many of the most commonly consumed food-accessories, answers a distinctly useful end. They serve, he maintains, the purpose of wholesomely slowing the otherwise too rapid digestion and absorption of copious meals. A too rapid digestion and absorption of food may be compared to feeding a fire with straw instead of slower burning coal. In the former case it would be necessary to feed often and little, and the process would be wasteful of the fuel; for the short-lived blaze would carry most of the heat up the chimney. To burn fuel economically, and to utilize the heat to the utmost, the fire must be damped down, so as to insure slow as well as complete combustion. So with human digestion; our highly prepared and highly cooked food requires, in the healthy and vigorous, that the digestive fire should be damped down, in order to insure the economical use of food. We render food by preparation as capable as possible of being completely exhausted of its nutrient properties; and, on the other hand, to prevent this nutrient matter from being wastefully hurried through the body, we make use of agents which abate the speed of digestion.

These remarks will apply, however, only to those who possess a healthy and active digestion. To the feeble and dyspeptic any food-accessory which adds to the labor and prolongs the time of digestion must be prejudicial; and it is a matter of common experience that beverages which in quantity retard digestion have to be avoided altogether by such persons, or partaken of very sparingly.—*Science.*

